# Flight and Walking in Locusts–Cholinergic Co-Activation, Temporal Coupling and Its Modulation by Biogenic Amines

**DOI:** 10.1371/journal.pone.0062899

**Published:** 2013-05-09

**Authors:** Jan Rillich, Paul A. Stevenson, Hans-Joachim Pflueger

**Affiliations:** 1 Institute for Neurobiology, Free University Berlin, Berlin, Germany; 2 Institute for Biology, University Leipzig, Leipzig, Germany; Mount Sinai School of Medicine, United States of America

## Abstract

Walking and flying in locusts are exemplary rhythmical behaviors generated by central pattern generators (CPG) that are tuned in intact animals by phasic sensory inputs. Although these two behaviors are mutually exclusive and controlled by independent CPGs, leg movements during flight can be coupled to the flight rhythm. To investigate potential central coupling between the underlying CPGs, we used the muscarinic agonist pilocarpine and the amines octopamine and tyramine to initiate fictive flight and walking in deafferented locust preparations. Our data illustrate that fictive walking is readily evoked by comparatively lower concentrations of pilocarpine, whereas higher concentrations are required to elicit fictive flight. Interestingly, fictive flight did not suppress fictive walking so that the two patterns were produced simultaneously. Frequently, leg motor units were temporally coupled to the flight rhythm, so that each spike in a step cycle volley occurred synchronously with wing motor units firing at flight rhythm frequency. Similarly, tyramine also induced fictive walking and flight, but mostly without any coupling between the two rhythms. Octopamine in contrast readily evoked fictive flight but generally failed to elicit fictive walking. Despite this, numerous leg motor units were recruited, whereby each was temporarily coupled to the flight rhythm. Our results support the notion that the CPGs for walking and flight are largely independent, but that coupling can be entrained by aminergic modulation. We speculate that octopamine biases the whole motor machinery of a locust to flight whereas tyramine primarily promotes walking.

## Introduction

Central pattern generators (CPG) are the basis of many ongoing repetitive movements and have been intensively studied on the cellular and network levels over the past two decades (reviews: [1]-[3]). The most influential finding emergent from work on CPGs is that each synapse in a network is a target to modulation by a wide variety of neuromodulators. This astonishing plasticity has led to the realization that an anatomically defined neuronal circuit has the potential to generate a wide variety of outputs, simply by modulating the degree of synaptic coupling between its individual elements.

Insect segmental ganglia possess several CPGs for generating the different motor patterns that underlie for example flight [4], [5], song production [6], [7], breathing movements [8], feeding movements [9], egg laying [10] and walking [11]. While most evidence suggests that each of these motor patterns are generated by separate and dedicated CPGs (e.g. [6], [12]), they need not be entirely independent. During flight, for example, the respiratory rhythm becomes reconfigured [13] and leg movements may be coupled to the wing-beat cycle [14]. Such effects could result from afferent control since in intact animals proprioceptive feedback plays an important role in tuning CPGs to generate functionally adequate motor activity [15]. In the locust flight system, for example, proprioceptors can register slight alterations in the contraction times of individual muscles and invoke a reset of the entire ongoing rhythm [16], [17]. In this paper we aim to reveal central interactions between the flight and walking CPGs of the locust *Schistocerca gregaria* by using aminergic and cholinergic agonists to elicit fictive motor patterns in deafferented preparations in which all sources of potential sensory feedback are eliminated.

Sombati and Hoyle [18] claimed that iontophoretic injection of octopamine into one of two discrete neuropil regions of a metathoracic ganglion could evoke either walking or flight. This was later confirmed for flight [5], but while octopamine can restore walking in hypokinetik, wasp-stung, cockroaches [19], little else is known about the role of octopamine in initiating walking. In particular it is unknown whether the two motor patterns, which are normally mutually exclusive, can occur simultaneously.

More recent data suggests that a cholinergic mechanism underlies the actual initiation of flight in locusts [20], while amines such as octopamine and tyramine are more likely to act as neuromodulators [21]–[24]. An analogous scenario is conceivable for walking and other insect CPGs. For example, and in addition to flight [20], topical application of the muscarinic agonist pilocarpine to insect ganglia has been shown to activate the CPG for walking [25], [26], song [27] and feeding [28]. But again it is not known whether e.g. pilocarpine induced fictive walking can occur at the same time as fictive flight.

Our data from the deafferented nervous system of locusts support the notion of two distinct CPGs for walking and flight, but with a variable degree of temporal coupling between the two depending on the action of biogenic amines. We speculate that octopamine biases the whole motor machinery of a locust to flight activity whereas tyramine’s major effect is more likely to be on the walking motor circuit.

## Materials and Methods

### Experimental Animals

All experiments were performed on adult male desert locusts (Schistocerca gregaria) from our own colony at Berlin maintained at a constant light/dark regime (12 h light: 12 h dark) at 30°C day/approx. 20°C night. All experiments complied with the Principles of Laboratory Animal Care and the German Law on the Protection of Animals (Deutsches Tierschutzgesetz).

### Preparation

**Figure 1:**
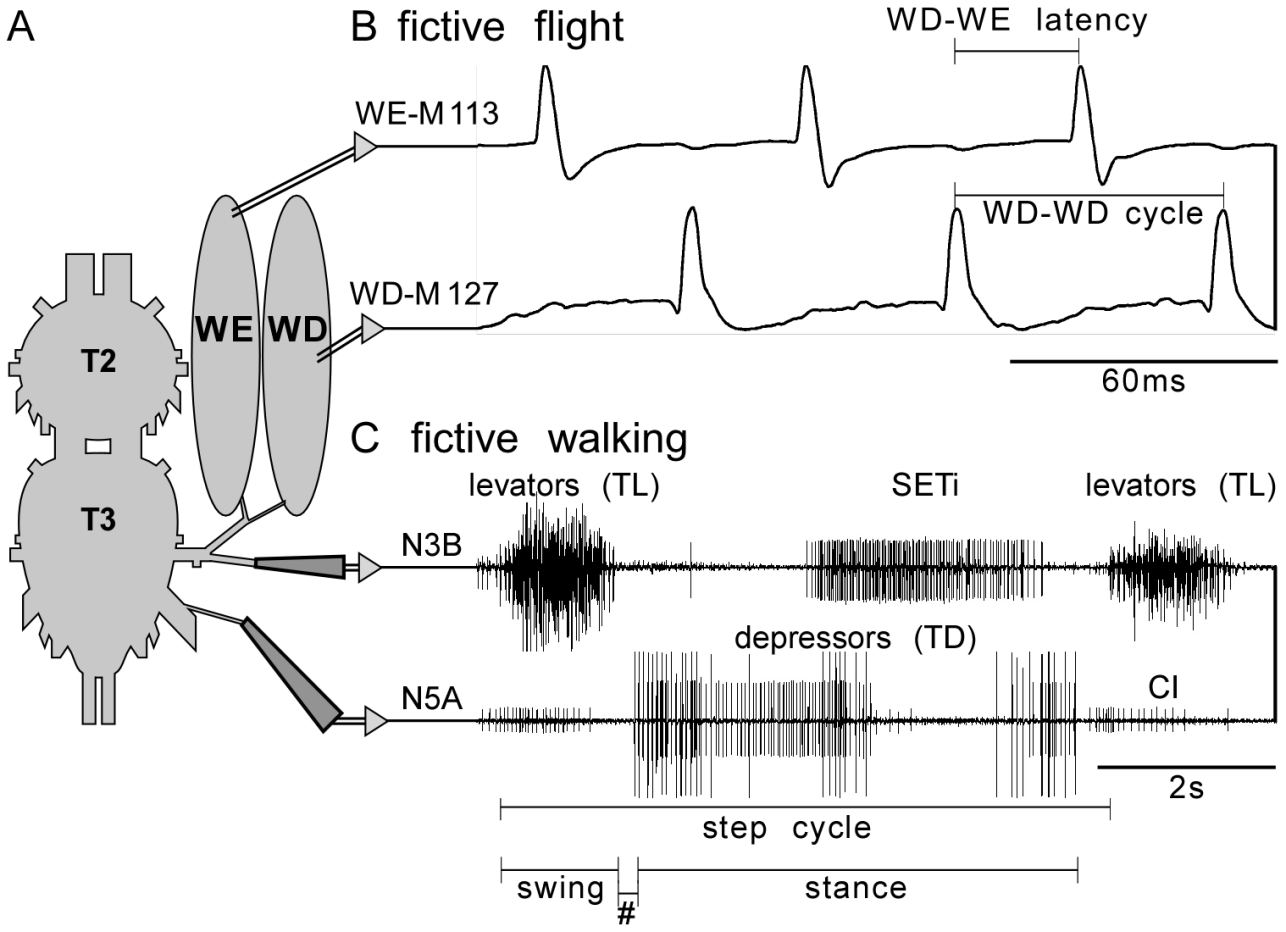
Schemata showing the standard preparation. **A** deafferented ganglia (T2 meso- T3 metathoracic) with intact nerves to a wing elevator and wing depressor muscle (WE-M113, WD-M127) and severed nerves to leg muscles: N3B, which carries seven trochanteral levator motoneurons (TL) that innervate muscle M131 and the slow extensor tibiae motoneuron (SETi) to muscle M135; N5A, through which the common inhibitor (CI) and slow and fast trochanteral depressor motoneurons project to innervate the trochanter depressor muscle (TD, M133A). **B** Electromyograms from WE-M113 and WD-M127 showing fictive flight induced by bath application of the muscarinic agonist pilocarpine. We evaluated the rhythm cycle length (WD-WD), the WD-WE latency and the phase of the elevator in the depressor cycle (WE/WD-WD - not indicated). **C** Nerve recordings from nerves N3B and N5A showing fictive walking evoked by pilocarpine. The TL units in N3B serve to monitor the swing phase and the TD units the stance phase. We measured step-cycle length (TL-TL), the length of the swing phase (TL burst length) and the TL-TD latency (#, end of TL burst to begin of TD activity). Further details in text.

Major features of the flight and walking motor pattern were analyzed in a deafferented meso-metathoracic ganglia preparation (T2–T3) as illustrated in Fig. 1. The animals were cooled for at least 10 min before any dissections were performed. For working with isolated and deafferented preparations, the locusts were decapitated followed by cutting off appendages, the pronotal shield and the abdomen posterior to the third abdominal segment. The remaining thorax was opened dorsally by cutting along the longitudinal midline, fixed ventral side down and super fused with locust saline containing (in mM): 150 NaCl, 5 KCl, 5 CaCl_2_, 2 MgCI_2_, 10 Hepes, 25 sucrose at pH 7.4. Air sacks and fatty tissues covering the ventral nerve cord were removed, whereas main tracheas that supply air to the nervous system were left intact. To abolish any sensory inputs to the central pattern generators, the ganglia were deafferented by cutting the connectives to the prothoracic ganglion and to all free abdominal ganglia. Furthermore, all peripheral nerve branches originating from the meso- and metathoracic ganglia were cut except nerve N3A of the metathoracic ganglion (numbered after [29]) that contains the motor axons of the recorded wing depressor and elevator muscles (M127, M113, numbered after [30]). This nerve is not known to innervate sense organs, although the existence of sensory axons cannot be entirely excluded [20].

In some experiments, natural initiation of flight was realized by supplying wind to head hairs with a commercial hairdryer. In this case the connectives between the head and the thoracic ganglia remained intact but all peripheral nerves of the prothoracic ganglia were severed as were those of the meso- and metathoracic ganglia excepting nerve N3A described above.

### Electrophysiological Recording

We used bipolar stainless steel wire electrodes insulated but for the tip (diameter 30 µm) to record electromyograms from hind wing elevator (WE, M113) and depressor (WD, M127) muscles, and custom made suction electrodes to monitor the activity from hind leg motor units in cut nerve N3B and N5A that are known to participate in the swing (N3B) and stance phase (N5A) of walking [25]. The recordings were amplified by a differential AC amplifier (model 1700, A-M Systems, Carlsborg, WA, USA), digitally converted by a CED Micro3 1401 and evaluated using the software spike 2 version 6.11 (Cambridge Instruments, Somerville, MA, USA).

### Pharmacological Treatments

Unless stated otherwise, all drugs were obtained from Sigma Aldrich (Deisenhofen, Germany). Cholinergic and aminergic agents were bath applied to the exposed thoracic ganglia to elicit walking and flight motor activity using a custom made perfusion system. DL-octopamine hydrochloride, tyramine hydrochloride and pilocarpine hydrochloride were dissolved in locust saline (see above) and the effective dosages were tested in a dilution series. Solutions of the tyramine receptor blocker yohimbine [31] or the selective octopamine receptor blocker epinastine [32] were used at a concentration of 1 mM dissolved in 0,1% aqueous dimethylsulphoxide (DMSO).

### Data Analysis

**Figure 2:**
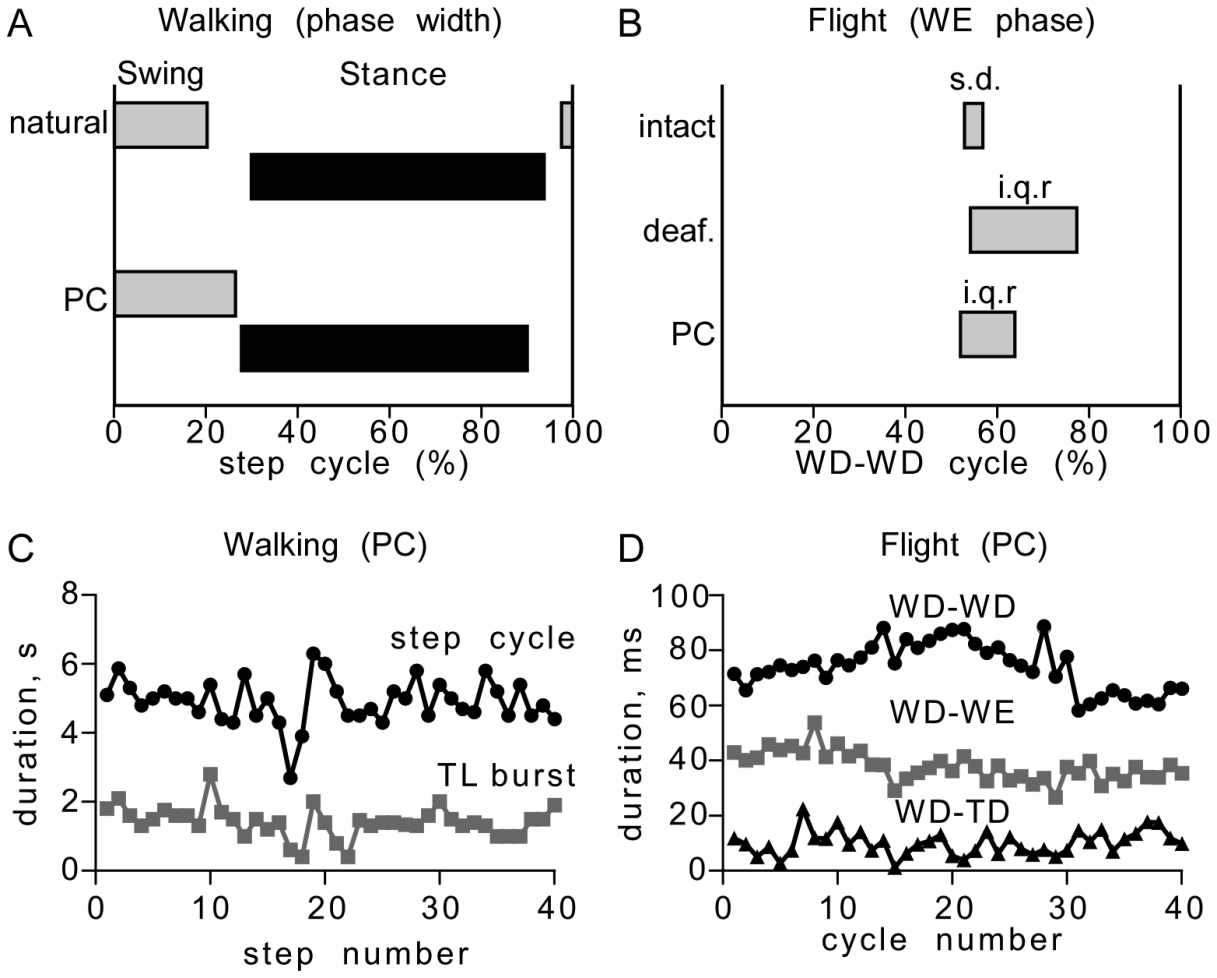
A general comparison of natural and pharmacologically induced walking and flight motor activity in intact and deafferented locusts. **A** Walking. Bars giving the start and end of the swing (grey) and stance (black) phases within the step cycle. Top (natural): data for naturally occurring, free walking from [34] (means of the activity of two antagonistic coxal muscles). Bottom (PC): Pilocarpine (1 mM) induced fictive walking (this paper, medians of 19 animals, 15 cycles each). **B** Flight. Bars giving the range of the phase of wing elevator (WE) units in the depressor cycle (WD-WD). Top (natural): Standard deviation (s.d.) of phase for intact tethered flying locusts from [35]. Middle (deaf.): Interquartile range (i.q.r.) of phase for wind-induce fictive flight of deafferented locusts (this paper, 35 animals, 100 cycles each). Bottom (PC): Interquartile range (i.q.r.) of phase for pilocarpine (1 mM) induced fictive flight of deafferented locusts (this paper, 19 animals, 100 cycles each). **C, D** Sequential plots comparing pilocarpine (PC, 1 mM) induced fictive walking and fictive flight respectively. **C** Step cycle length (black circles) and duration of the TL burst (grey circles) for 40 step cycles over a 3 min period. **D** Wing depressor cycle length (WD-WD, black circles), wing depressor-elevator latency (WD-WE, grey squares) and wing depressor-trochanteral depressor latency (WD-TD, black triangles) for 40 fictive flight cycles over a 3 s interval. Note that the flight sequence analyzed here comprises an entire stance phase of simultaneously occurring fictive walking. Abbreviations as in Fig. 1.

To evaluate the fictive walking pattern we measured the following parameters for 15 step cycles of each individual animal evaluated using the cursor function of recording software: step cycle length, burst length of the levator trochanteris (TL) muscle, latency between end of the TL burst and onset of trochanteral depressor (TD) muscle activation (TL-TD latency; c.f. Fig. 1). These 15 cycles were found to be sufficiently large to represent the activity recorded for a single animal (Fig 2C). To analyze flight activity we used the standard threshold spike detection mode of the software spike 2. Flight muscle activity was evaluated from 100 consecutive fictive flight cycles for each preparation. As depicted in Fig. 1, we evaluated the cycle length as the time between two consecutive hind-wing depressor muscle (WD, M127: WD-WD) bursts, the latency between WD M127 and hind wing elevator (WE) muscle M113 (WD-WE) and finally the phase of the elevator muscle in the depressor cycle (WD-WE/WD-WD). The coupling of walking activity to the flight rhythm was evaluated from the WD-TD latency and the phase of TD in the depressor cycle (WD-TD/WD-WD).

In wind induced flight the coupling of TD to the wing beat cycle strongly varied in its degree. Thus, to compare the impact of amines and their receptor blocker before and after pharmacological treatments, we normalized all data of each preparation to the median of latency respectively phase of the pre-treatment condition (vehicle).

The median and the interquartile range (IQR) of latency and phase relationship were calculated for non-parametric data sets. To allow comparisons with earlier studies [5] [20] we employed similar standard linear statistical analysis using standard commercial software (Prism 5, Graph Pad Software Inc. La Jolla, CA, USA).

The degree of coupling between of WE and TD to WD under different pharmacological treatments can be assessed from the interquartile range (the difference between the third quartile and the first quartile), which is a common and robust measure of statistical dispersal [33], whereby a greater IQR indicates a lower degree of coupling. In wind induced flight we compared the relative change of the IQRs before and 30 min after pharmacological treatments for each preparation.

The significance of differences in distributions was tested by the Mann Whitney U test for unpaired data and by the Wilcoxon Signed Rank test for paired data. The Chi-square test was employed for comparing relative frequencies. Charts and pictograms were edited using a standard graphic program (Canvas X, Deneba Systems Inc.).

## Results

The natural motor patterns for walking and flight are readily distinguishable by their temporal components (Fig. 2). Natural walking in intact freely behaving locusts is characterized by a burst of swing phase motor units, followed after a delay by a longer burst of stance phase units, whereby the swing phase is the shorter and occupies about 20% of a step cycle (Fig. 2A), which varies in intact animals from 200–1300 ms [34]. For tethered flight of intact locusts, individual motor units are usually activated only once or twice per cycle, whereby the wing elevator and wing depressor units fire in alternation and the WD-WE latency is generally 55% of the total cycle length (Fig. 2B) which lasts some 50 ms, but 80 or more ms in deafferented preparations [35].

### Fictive Walking and Flight in a Deafferented Nervous System

The muscarinic agonist pilocarpine and the biogenic amine octopamine are known to initiate fictive walking (pilocarpine: [25]; octopamine: [18]) and fictive flight (pilocarpine: [20]; octopamine: [18], [34]) in deafferented locust nervous systems that matches the natural motor patterns albeit at a slower rhythm frequency (Fig. 2). In a first set of experiments we aimed to analyze whether the central pattern generators (CPGs) for walking and flight can be co-activated in a deafferented nervous system (Fig. 1) and to what extent their motor outputs influence each other. As given in detail below, we found the probability of a given drug evoking a specific motor pattern was dose dependent, although the actual dose required varied from preparation to preparation. With respect to details of the pattern elicited, however, there was no obvious dose-dependent differences.

#### Pilocarpine

**Figure 3:**
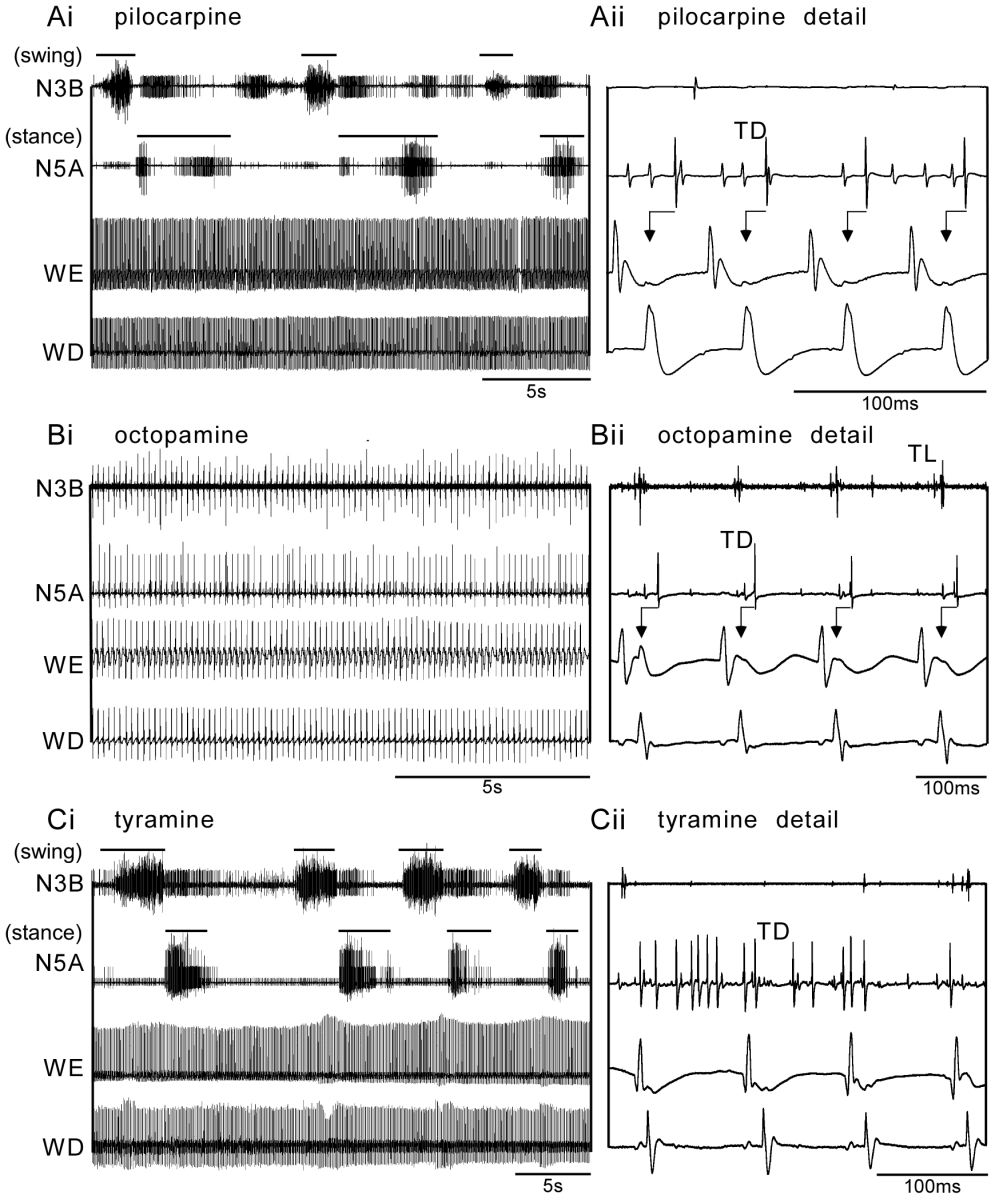
Exemplary simultaneous recordings showing motor activity in leg (upper two traces) and wing motor units (lower two traces) evoked by drug application in deafferented locust preparations: A pilocarpine (1 mM), B octopamine (100 mM), C tyramine (100 mM); i, ii each show a longer sequence and detail respectively. Abbreviations as in Fig. 1. Note that at the given concentrations pilocarpine and tyramine usually both evoke fictive walking together with flight, while octopamine evokes fictive flight in wing and leg motor units. The swing and stance phases of fictive walking are indicated by bars in **Ai** and **Ci**. Note coupling of leg unit TD to the WD (arrows in **Aii**, **Bii**).

Applied at low concentrations, pilocarpine usually elicited a fictive walking pattern (0.1 mM, 75% of n = 12 preparations), whereas higher dosages were required to induce a fictive flight pattern in the deafferented nervous system (1 mM, 70%, n = 27; 5 mM, 78%, n = 9; Fig. 3Ai, 4Ai and Aii). Interestingly, fictive flight activity did not suppress walking motor activity so that at a concentration of 1 mM the two motor patterns were produced simultaneously in 70% of all preparations. Fig. 3Ai illustrates a recording in which both motor patterns are shown simultaneously in the respective output elements. The walking CPG generally started to produce coordinated output within 1 min of drug application, whereas flight activity mostly followed with a delay of 1 to 3 min. Both motor patterns then occurred simultaneously and maintained their basic temporal pattern (for details see below). We observed no indication of mutual inhibition, such as alternating sequences of activity of the two CPGs, and no obvious changes in the basic swing-stance rhythm when fictive flight commenced. Nevertheless, the flight CPG exerts a clear influence on the walking CPG since at least one of the observed leg motoneurons (TL, fast TD) were frequently temporally coupled to the flight rhythm, so that each spike in a step cycle occurred synchronously with WD firing at flight rhythm frequency (Fig. 3Aii).

#### Octopamine

Whereas pilocarpine elicited walking and flight, octopamine only rarely evoked a walking pattern in the observed leg motor units (Fig. 3B). Walking activity occurred in only 13% of all cases using a concentration of 100 mM (n = 23, Fig. 3Bi, 4B). However, octopamine very effectively evoked flight at this high dosage (91%, Fig. 3Bii), and almost all co-activated leg motor units recorded were coupled to the flight rhythm throughout the whole sequence (cf. Fig. 3Bii).

#### Tyramine

**Figure 4:**
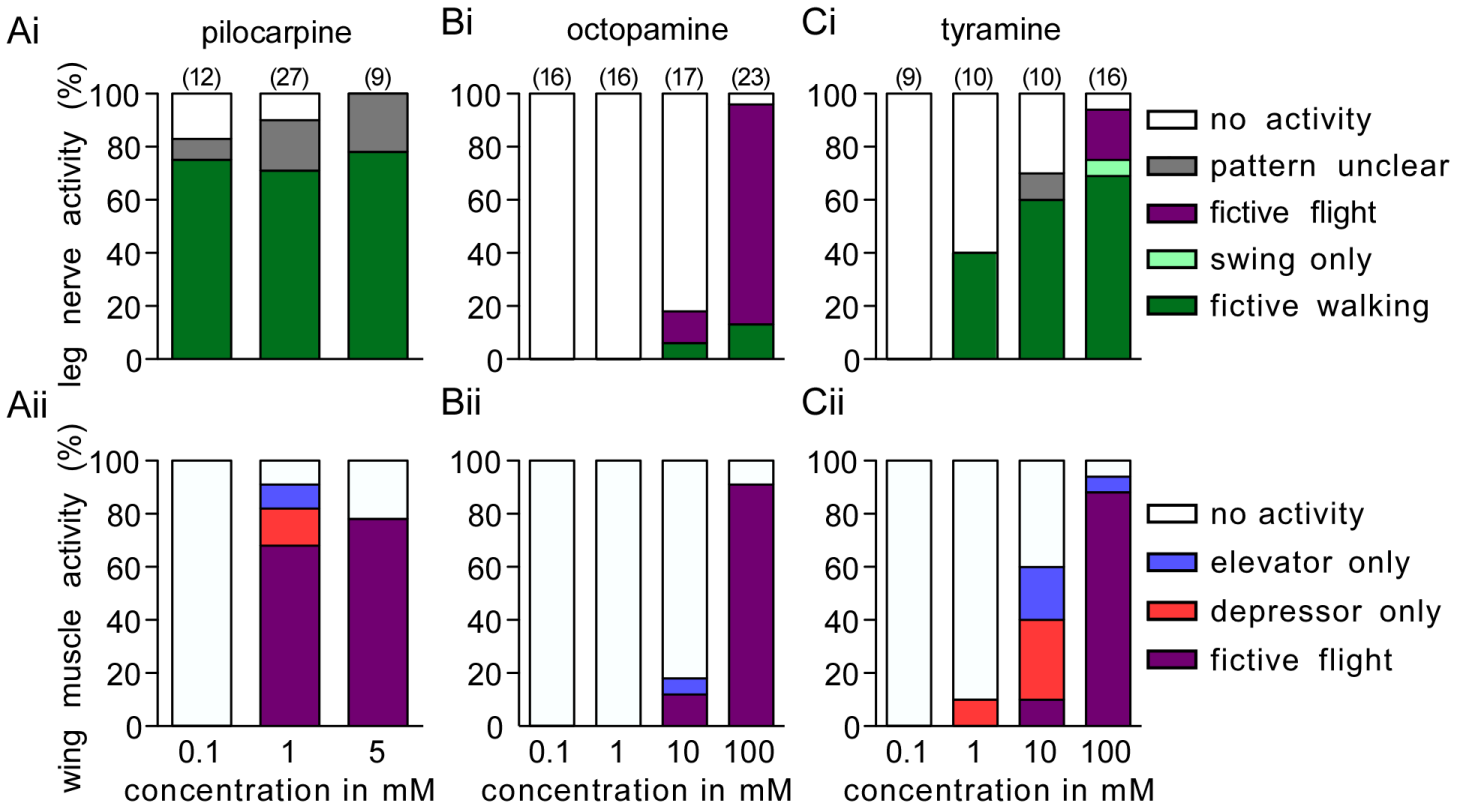
Bar charts giving the concentration dependent occurrence (%) of different types of motor activity evoked by: A pilocarpine, B octopamine, C tyramine in leg nerves (i upper charts) and wing muscles (ii lower charts). Key (leg nerves): white bars: no activity; grey bars: pattern unclear; purple bars: fictive flight; light green bars: swing phase only; dark green bars: fictive walking. Key (wing muscles): white bars: no activity; blue bars: elevator only; red bars: depressor only, purple bars: fictive flight activity. Numbers in parenthesis give the number of preparations for each test group.

In contrast to octopamine, its precursor tyramine evoked a fictive walking pattern at comparatively low concentrations (10 mM: 60% of n = 10 preparations, Fig. 4Ci), but a 10 fold higher dosage was required to elicit fictive flight (100 mM, 88%, n = 16, Fig. 3Ci, 4Cii). The capacity of tyramine to induce fictive walking was significantly higher than that of octopamine even at a concentration of 100 mM (69%, n = 16; Fig. 4Cii; Chi-square, p<0.001, Chi-value, 12.8, degrees of freedom, df 1). As found for pilocarpine, both motor patterns could occur simultaneously in response to tyramine application (69% of all cases) without change in the basic temporal features of each pattern (Fig. 3Ci). However, in contrast to pilocarpine, tyramine induced fictive flight had only a weak effect on the walking motor pattern, since coupling of leg units to the flight rhythm was rarely observed, and whenever it occurred the latency of wing to leg motor units was far more variable (Fig. 3Cii, further details below).

### Analysis of the Walking Pattern

Whenever fictive walking was induced by pilocarpine or by tyramine the patterned activity persisted for twenty or more minutes, without any obvious perturbations (see for example Fig. 2C, which shows a sequential plot of pattern parameters for a 3 min period). Our evaluation was restricted to a representative sequence of 15 step cycles for each animal.

**Figure 5:**
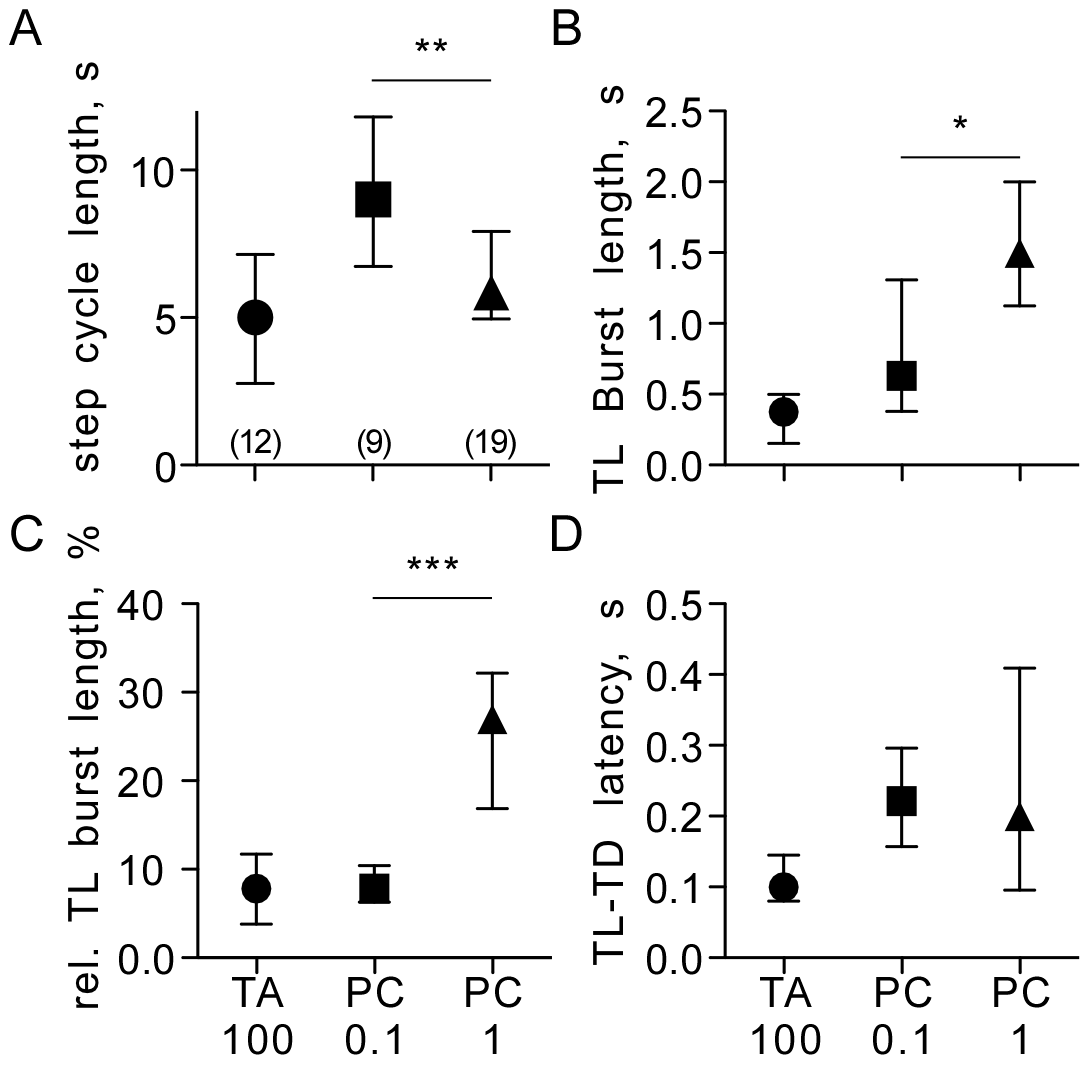
Charts comparing key features of fictive walking patterns released by tyramine (TA, circles: 100 mM) and by a low and higher concentration of pilocarpine (PC, squares: 0.1 mM, triangles: 1 mM). A Step cycle length, B TL burst length, C relative TL burst length (as % of step cycle length), D TL-TD latency. Symbols give the median, ticks the interquartile range and asterisks significant differences between indicated groups (Mann Whitney U test for unpaired data sets *p<0.05, **p<0.01, ***p<0.001). Numbers in parenthesis (chart A) give the number of preparations for each test group.

For pilocarpine (0.1 mM) induced fictive walking, the step cycle lasted between 6.9–11.1 s (IQR, median 9.1, n = 9 preparation, 15 cycles each, Fig. 5). The swing phase defined by the duration of the levator burst (TL, up to seven motoneurons) lasted 0.63 s (median, IQR 0.41–1.05) so that the relative burst length in a step approximately amounted to 8% (median, IQR 6–9). The stance phase defined by the activity of the slow and fast depressors of the trochanter (TD) subsequently followed with a latency of 0.22 s (median TL-TD latency, IQR 0.16–0.27).

A 10 fold higher pilocarpine dosage (1 mM) resulted in a significant reduction in step cycle length (median 5.85 s, IQR 5–7.4, U test, p<0.05, n = 19), and a increase in TL burst length (1.5 s, median, IQR 1.2–2.0, U test, p<0.05) and the relative burst length in a step (28%, median, IQR 18–36, U test, p<0.001), whereas the TL-TD latency was unaffected and remained constant at 0.2 s (median, IQR 0.1–0.32).

Tyramine (100 mM) induced walking had a step cycle duration of 5 s (median, IQR 2.9–6.1, at 100 mM, n = 12), a TL burst duration of 0.38 s (median, IQR 0.19–0.5), and the relative TL burst duration in a step amounts to 8.2% (IQR 4–11). All three measurements were within the range of those observed for at least one of the tested pilocarpine concentrations that induced fictive walking patterns, whereas the TL-TD latency was shorter than in both tested pilocarpine concentrations (median 0.1 s, IQR 0.08–0.14; 0.1 mM: U test, p<0.01; 1 mM: U test, p<0.05).

### Analysis of Flight Motor Activity

Whenever fictive flight was induced by pilocarpine, octopamine or tyramine the patterned activity persisted for several minutes, without any obvious perturbations (see for example Fig. 2D, which shows a sequential plot of pattern parameters for a 3 s period). Our evaluation was restricted to a representative sequence of 100 wing beat cycles for each animal.

**Figure 6:**
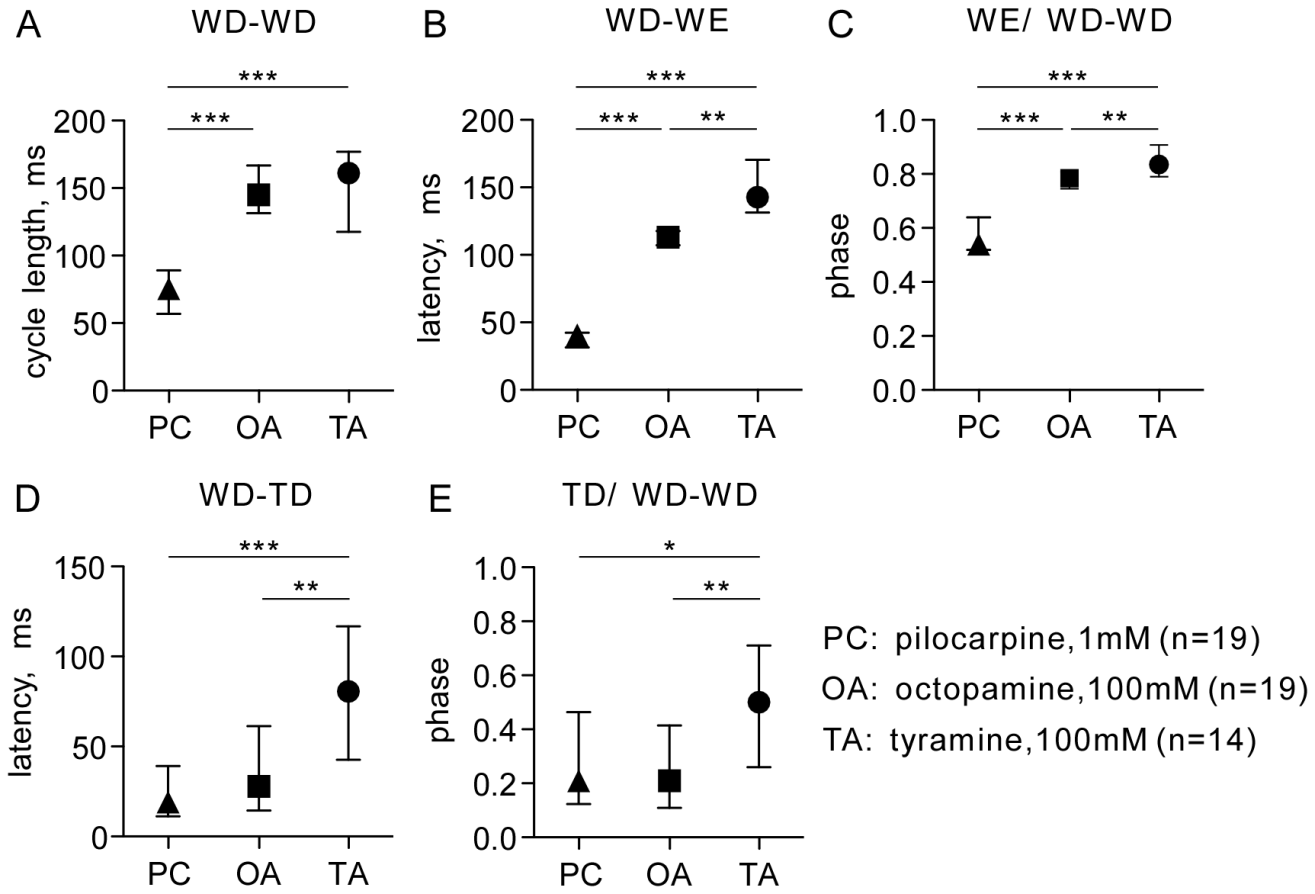
Charts comparing key features of fictive flight patterns released by pilocarpine (PC, triangles: 1 mM), octopamine (OA, squares: 100 mM) and tyramine (TA, circles: 100 mM). **A** Flight rhythm cycle length (WD-WD), **B** WD-WE latency, **C** phase of wing elevator in wing depressor cycle (WE/WD-WD), D WD-TD latency (coupling of leg unit TD to the wing unit WD), E phase of leg unit in wing depressor cycle (TD/WD-WD). Symbols give the median, ticks the interquartile range and asterisks significant differences between indicated groups (Mann Whitney U test for unpaired data sets *p<0.05, **p<0.01, ***p<0.001); n in the key gives numbers of preparations for each group evaluated.

Similar to findings of Buhl et al. [20], bath application of pilocarpine (1 mM) induced fictive flight with a cycle duration of 75 ms (median, IQR 59–85, n = 19 preparations, 100 cycles each; Figs. 6A–C). Depressor and elevator muscles were activated alternately with a WD-WE latency of some 40 ms (median, IQR 32–43), and elevator phase in the depressor cycle of 0.54 (median, IQR 0.52–0.64).

Octopamine (100 mM) evoked fictive flight patterns that were slower than those elicited by pilocarpine (flight cycle duration: median 144 ms, IQR 134–164, n = 19, U test, p<0.001).These patterns had a comparatively long WD-WE latency (median 113 ms, IQR 107–119, U test, p<0.001) as well as a shifted phase relationships (median 0.79, IQR 0.75–0.81, U test, p<0.001).

For tyramine (100 mM), the cycle duration of induced fictive flight pattern was similar to that of octopamine (median 161 ms, IQR 119–175) but the WD-WE latency (median 143 ms, IQR 132–171) and phase relationship (median 0.84, IQR 0.79–0.91) were both significantly greater (both parameters: U test, p<0.01).

**Figure 7:**
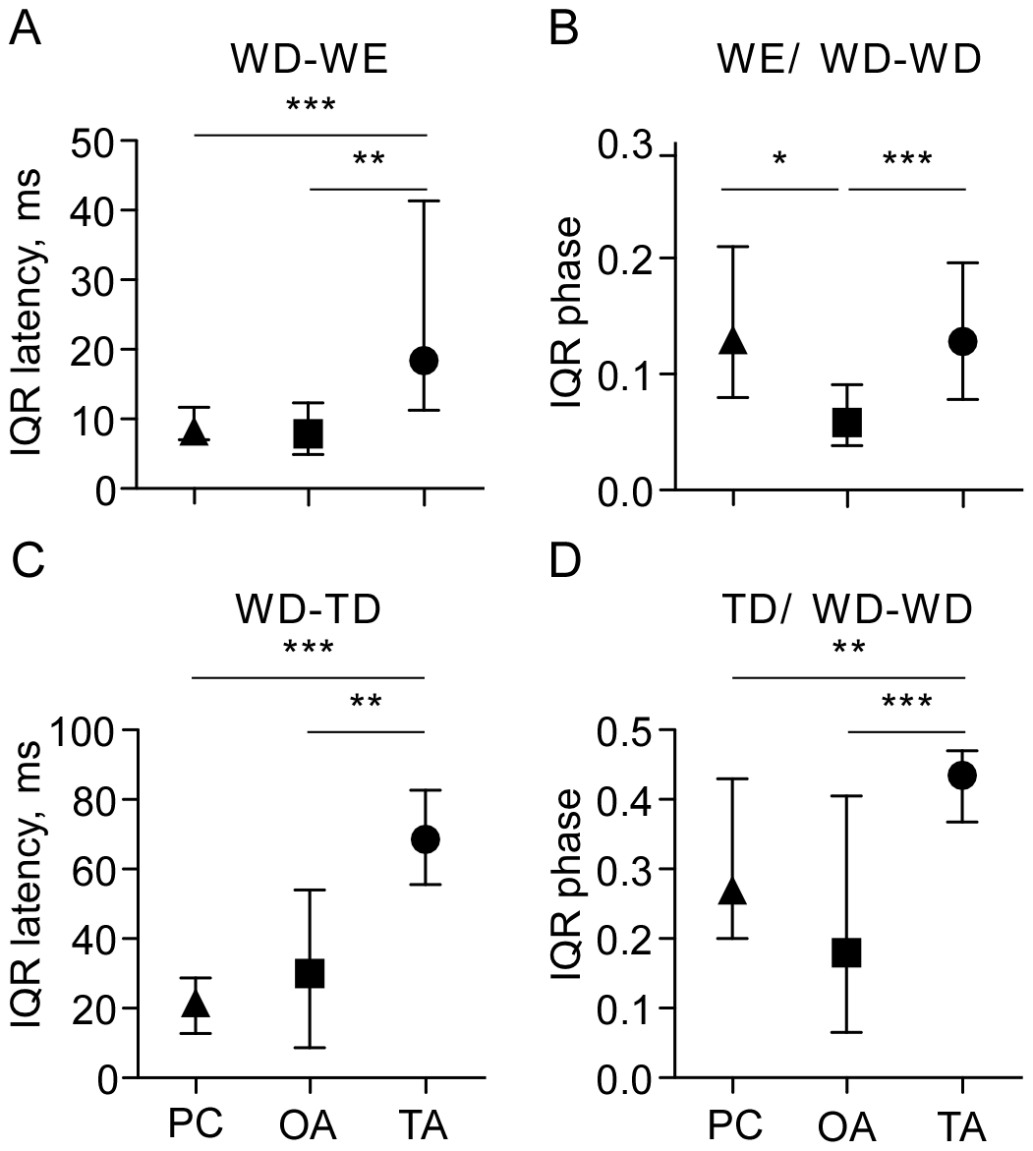
Charts comparing the degree of temporal coupling between different motor units for activity evoked by pilocarpine (PC, triangles: 1 mM), octopamine (OA, squares: 100 mM) and tyramine (TA, circles: 100 mM). Temporal coupling was evaluated from the statistical dispersal for non-parametric data sets as given by the interquartile ranges, IQR, of the latencies and phases between units recorded for each animal in a group [33]: **A** WD-WE latency, **B** phase of wing elevator in wing depressor cycle (WE/WD-WD), **C** WD-TD latency (coupling of leg unit TD to the wing unit WD), **D** phase of leg unit in wing depressor cycle (TD/WD-WD). Abbreviations as in Fig. 1. Symbols give the median (of the IQRs), ticks the IQR (of this median) and asterisks significant differences between indicated groups (Mann Whitney U test for unpaired data sets *p<0.05, **p<0.01, ***p<0.001).

**Figure 8:**
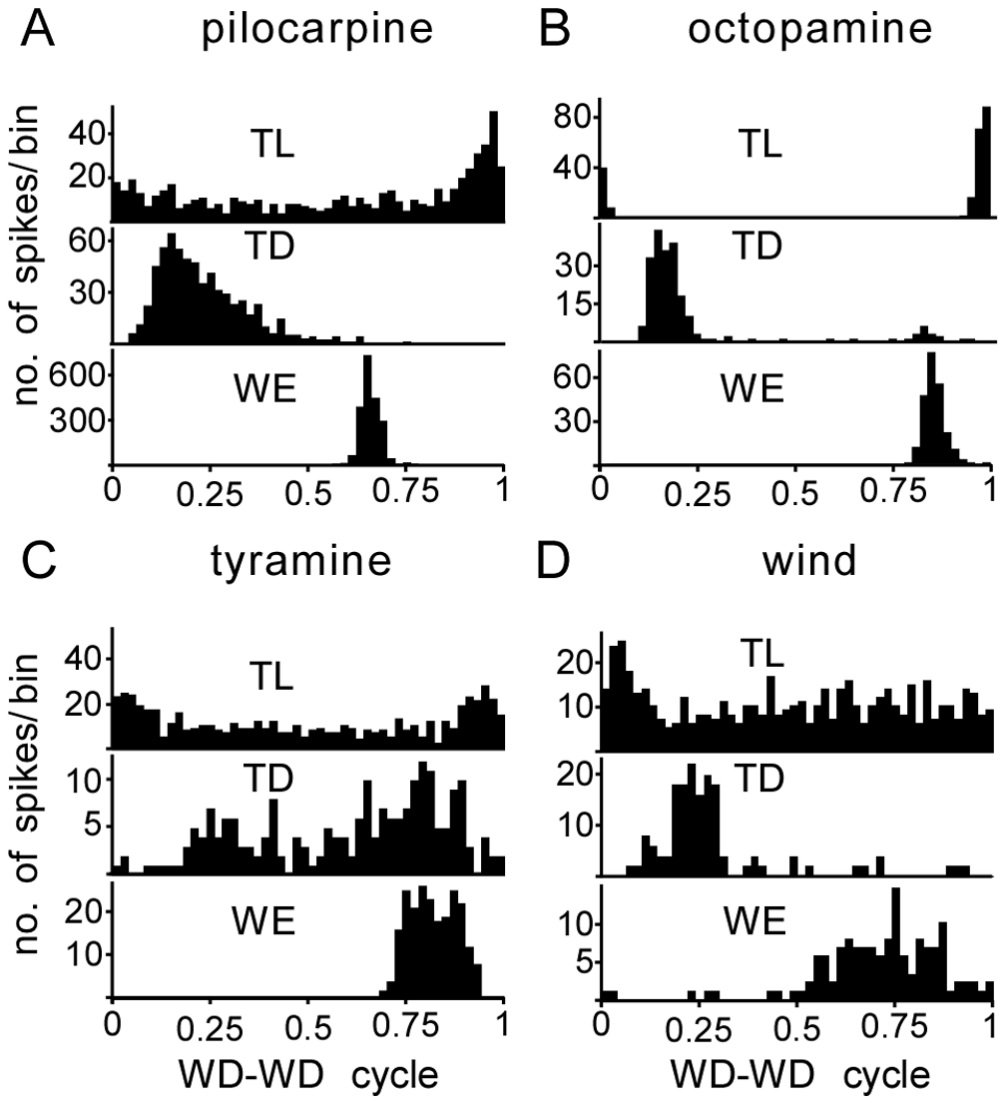
Phase histograms giving the timing of different motor units (TL, TD, WE) within the cycle length for a representative recording of the flight rhythm (WD-WD cycle, normalized) as evoked by A pilocarpine (1 mM), B octopamine (100 mM), C tyramine (100 mM) and D wind: TL (upper trace in each panel), TD (second trace) and WE (third trace) in a normalized WD-WD cycle. Each bin gives the number of spikes occurring in 1/50th of the cycle length.

To assess the degree of coupling of the timing of elevator muscle activation to depressor muscle activation we compared IQRs of the WD-WE latency and phase of the elevator in the depressor cycle for fictive flight sequences induced by pilocarpine, octopamine and tyramine (Fig. 7A, B; Fig. 8). This revealed that the IQRs of latency for the octopamine and pilocarpine-induced rhythms were significantly less than for tyramine-induced flight, indicating a higher degree of elevator-depressor coupling (WD-WE latency: U tests, p<0.01 for each, Fig. 7A). With respect to the phase, however, this was less variable for octopamine induced flight than for pilocarpine- (U test, p<0.05) and tyramine induced flight (U test, p<0.001; Fig. 7B).

### Coupling of Leg Motor Units to the Wing Beat Cycle

As shown for pilocarpine-induced activity (Fig. 3Ai), leg muscle motoneurons were primarily active in the walking pattern. In most cases, the fast TD unit (Fig. 3Aii and Fig. 7A second trace) and the TL units (Fig. 3Aii and Fig. 8A upper trace) were coupled to the flight rhythm, such that each spike in a step cycle occurred synchronously with wing depressor units firing at flight rhythm frequency. The coupling of the walking activity to the flight rhythm was evaluated from the WD-TD latency (median, 17.5 ms, IQR 11–39, n = 19 preparations, 50 cycles each, Fig. 6D) and the phase of the TD spikes in the depressor cycle (median 0.2, IQR 0.12–0.46, Fig. 6E).

Although, octopamine rarely elicited a clear walking pattern (Fig. 3Bi), the TD unit was activated during and coupled to flight motor activity throughout the entire sequences recorded (n = 19, Figs. 3Bii and 8B). The WD-TD latency (median 28 ms, IQR 14–61, n = 19, Fig. 6D) and phase (median 0.2, IQR 0.11–0.42, Fig. 6E) were not statistically different to those of pilocarpine-induced patterns. With respect to tyramine induced activity, we observed a weaker coupling of the TD to flight, which could occur at any time between 4–117 ms after the depressor (IQR of WD-TD latency, median 82 ms; median phase 0.5, IQR 0.26–0.71, n = 14 preparations, Figs. 6 and 8C).

The degree of WD-TD coupling was again assessed by comparing the IQRs. As shown in Fig. 7, the IQRs of WD-TD latency for pilocarpine and octopamine induced rhythms were both small compared to tyramine induced rhythms (U tests p<0.001, respectively 0.01; Fig. 7C). A similar trend was also evident for the TD phase (Fig. 7D).

### Wind Induced Flight

**Figure 9:**
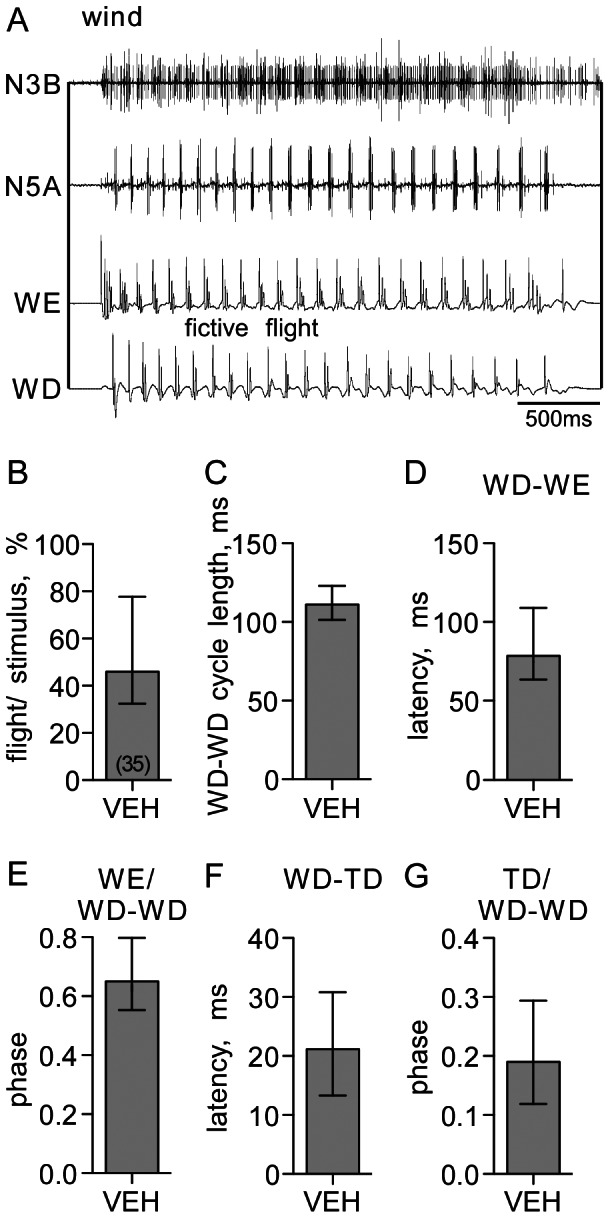
Evaluation of wind-evoked fictive flight. **A** Simultaneous extracellular recordings of leg and wing motor units in a deafferented locust preparations with an intact head (abbreviations as in Fig. 1). **B** Bar giving the effectiveness of the wind stimulus as assessed from the percentage (%) of evoked flight sequences recorded for 35 animals, each in response to a series of 10 successive wind stimuli. **C** WD-WD cycle length. **D** WD-WE latency in ms. **E** phase of wing elevator in wing depressor cycle (WE/WD-WD). **F** WD-TD latency. **G** phase of leg unit in wing depressor cycle (TD/WD-WD). Columns in **B–G** give the median, and ticks the IQR.

Flight is naturally released by a wind stream directed to head hairs in the absence of tarsal contact. Fig. 9A shows wind-induced fictive flight in a deafferented locust preparation with the exception of the head left intact and attached to the thoracic ganglia, but otherwise not different to our standard preparation. Since wind often failed to evoke any form of activity in these preparations, we evaluated the effectiveness of the wind stimulus by recording the responses of 35 locust preparation to a series of 10 successive wind stimuli (duration 5 s, interval 30 s). Each preparation exhibited flight activity at least once, and fictive flight was evoked in 46% of all trials (median, IQR 32–77, n = 35×10; Fig. 9B). For wind-induced fictive flight, the cycle length ranged from 103–118 ms (IQR, median 112 ms; 100 cycles evaluated for 1 sequence of each of the 35 preparations; Fig. 9C) and the WD-WE latency ranged from 67–113 ms (median 78, Fig 9D), with a phase of the WE in the WD-WD cycle of 0.65 (median, IQR 0.55–0.79, Fig. 9E). As for pharmacological induced flight, we often observed tight temporal coupling of the TD to the wing beat cycle (WD-TD latency: median 21 ms, IQR 13–31; TD phase: median 0.19, IQR 0.12–0.29; Fig. 8D and 9F, G).

**Figure 10:**
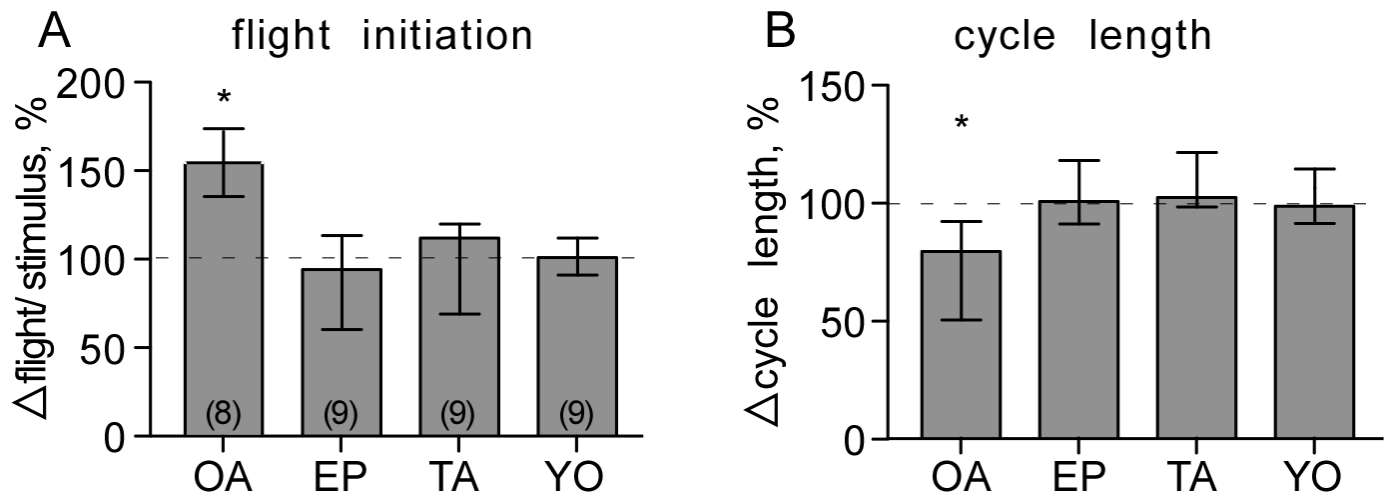
Relative changes (%) in A the effectiveness of wind stimulation to initiate flight, and B cycle length (WD-WD) of wind evoked fictive flight after treatment with (from left to right): octopamine (OA, 5 mM), the octopamine receptor antagonist epinastine (EP, 1 mM), tyramine (TA, 5 mM) and the tyramine receptor antagonist yohimbine (YO, 1 mM). Numbers in parenthesis (in **A**) give the number of evaluated preparations for each test group. Columns give the median, ticks the interquartile range and asterisks significant changes (Wilcoxon Signed Rank test *p<0. 05).31.

The probability of inducing flight by wind was significantly increased in the presence of bath applied octopamine (5 mM; Wilcoxon Signed Rank test, p<0.05; Fig. 10A), whereby the cycle length of the rhythm was significantly shorter (Wilcoxon Signed Rank test, p<0.05; Fig. 10B). Contrasting this, neither the probability of inducing flight by wind, nor the cycle length of the evoked rhythm was affected by tyramine (5 mM; Fig. 10A, B). Similarly, application of the corresponding amine receptor blockers epinastine and yohimbine had no effect on these two parameters (both 1 mM; Fig. 10A, B).

**Figure 11:**
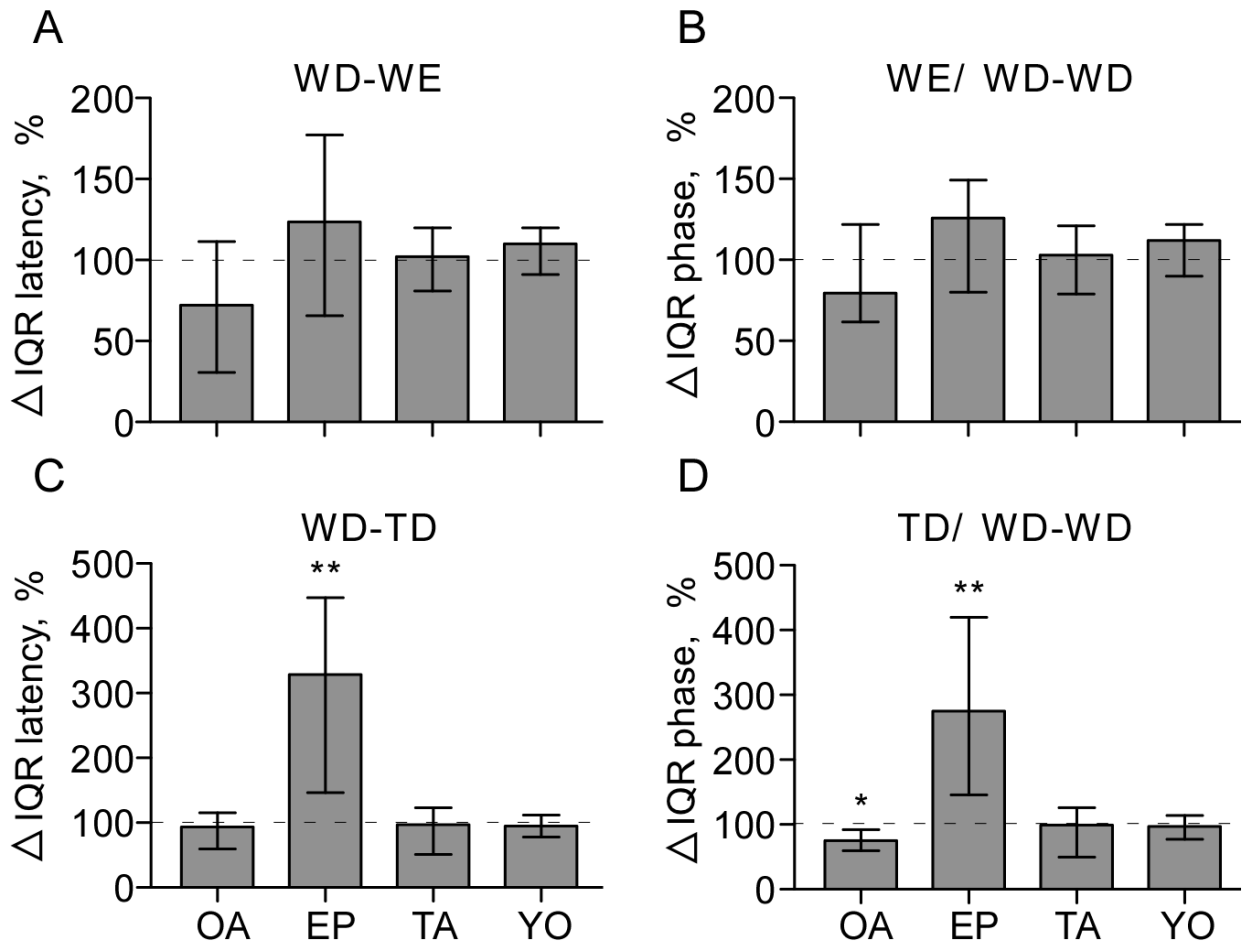
Relative changes (%) in the degree of temporal coupling between different motor units (evaluated as in Fig. 6) for wind evoked fictive flight following treatment with (from left to right): octopamine (OA, 5 mM), the octopamine receptor antagonist epinastine (EP, 1 mM), tyramine (TA, 5 mM) and the tyramine receptor antagonist yohimbine (YO, 1 mM). Data from same preparations as for Fig. 9. **A** WD-WE latency, **B** phase of wing elevator in wing depressor cycle (WE/WD-WD), **C** WD-TD latency (coupling of leg unit TD to the wing unit WD), **D** phase of leg unit in wing depressor cycle (TD/WD-WD). Columns give the median, ticks the IQR and asterisks significant differences (Wilcoxon Signed Rank test *p<0.05, **p<0.01).

The strength of coupling between wing motor units (WD-DE latency and WD phase) and leg motor units to the flight rhythm (WD-TD latency and TD phase), was assessed by comparing the relative changes in IQRs of these parameters before and after pharmacological treatments for each preparation (Δ IQR, %). While the degree of coupling of the WE to the WD (Fig. 11A) and the WD phase (Fig. 11B) were both unaffected by octopamine, tyramine or their antagonists, the coupling of the fast TD unit to the wing depressor (WD), and the TD phase in the depressor cycle, became significantly less tight after applying epinastine (Wilcoxon Signed Rank test, p<0.01 in both cases; Fig. 11C, D). Octopamine, on the other hand, had an opposing effect, i.e. the TD tended to become more tightly coupled to the flight rhythm (Fig. 11D; Wilcoxon Signed Rank test, p<0.05), while tyramine and its antagonist had no influence.

## Discussion

### Co-activation of the CPGs for Walking and Flight

Activation of the CPG for locust flight is dependent on a cholinergic mechanism [20], and the same is likely to apply to the CPG for walking in locusts [25], stick insects [26] and adult moths [36]. Our finding that these two CPGs can operate at the same time is surprising considering that the behaviors they generate are mutually exclusive under normal conditions. This results neatly illustrates that both the activation pathways and core generating circuitry must be functionally and anatomically separate in the two systems. As much has already been postulated on the grounds that the key rhythm generating interneurons so far identified are different for each of the two networks [12], and separate neurons leading to activation of flight and walking have been identified (review: [37]). However, in the light of the frequently neglected finding, that hemi-sected locust ganglia can generate a near normal flight [38], it could be argued that the “key rhythm generating interneurons” have not yet been identified. Clearly, our data suggest that there are unlikely to be any individual neurons that play a major part in generating both patterns. Since the walking pattern, which was usually established first, was not affected by the onset of fictive flight, and visa versa, it also seems unlikely that mutual exclusivity of the two behaviors is promoted by cross inhibitory interactions. Hence, while reciprocal central inhibitory networks underlie pattern generation in the flight CPG [12], there appears to be no inter-network suppression at this level. Under natural conditions sensory feedback mechanisms, such as the “tarsal reflex” [39], along with specific natural releasing stimuli, seem rather to ensure selective recruitment of behaviorally adaptive motor performances.

### Temporal Coupling between the CPGs for Walking and Flight

Distinct but interacting motor circuits are often observed in invertebrates [40]. In orthopterans, respiration co-occurs with a variety of other motor patterns, that can influence each other (e.g. stridulation: [41]; foregut rhythms: [42]; walking: [25]; flight: [13]). Similarly, and although in essence separate, we found clear interactions between the CPGs for flight and walking. During flight of intact locusts, leg movements and some leg motor units may be coupled to the wing-beat cycle [14]. Although this could be controlled by sensory feedback [43], we found evidence for central nervous interactions. During pharmacologically induced fictive flight of fully deafferented locusts, selected leg motor units, that are active during the stance phase (TD), fire in close phase with wing depressor motor units firing at flight frequency, but remain silent during the swing phase. Contrasting this, temporal changes in the ongoing flight pattern are not associated with the onset or termination of fictive walking, or with the swing and stance phases. Although flight neurons do receive inputs from the respiratory rhythm generating circuits [44], there is no evidence that dedicated flight neurons are driven by elements of the walking circuitry, with the exception perhaps of motoneurons that innervate bifunctional muscles ([12]; see however [34]).

But why should leg motor units be recruited in phase with the flight rhythm? The investigated trochanteral depressor (TD) leg muscle is normally active in the stance phase during walking of unrestrained locusts, and has hitherto not been noted as a bifunctional muscle involved in flight [45], [12] or hindleg steering during flight [46]. Since the TD motor units are also recruited during wind-induced flight, in a similar way described for coxal muscles during steering maneuvers [46], [14], we suspect that numerous other leg muscles are probably activated during flight and that the TD muscles may be involved in flight steering. Alternatively, centrally mediated coupling of leg muscle activation to the flight rhythm may ensure that passive perturbations of the legs during flight resonate in tune with flight, and thereby mechanically stabilize the flying insect.

### Aminergic Modulation of the CPGs for Walking and Flight

Several authors report that fictive flight motor activity can be evoked in insects by treatment with octopamine (locusts: [18], [35], [47], [48], [20]; moths: [49]; [50]), tyramine (locusts: [20]) and dopamine (locusts: [20]; moths: [49]) but not serotonin ([20]; cf. however [51] on Drosophila). Despite this, none of the flight initiating neurons that have been identified appears to be aminergic [52]. Furthermore, and in contrast to acetylcholine (cf. [20]), experiments using amine depleting agents, receptor blockers or genetic knock-down show that amines are not essential for flight initiation (crickets: [53]; fruit flies: [54]; locusts: [20]). This does not however exclude a role of amines in controlling the expression of flight behavior. Multiple transmitter systems are known to have a profound impact on production of motor behavior, in both vertebrates and other invertebrates (reviews: [55], [3]), and amines in particular are equated with a key role in modulating the activity and operation of CPGs (for a reviews on locusts see: [56], [22], [2]). Our data illustrate that the amines octopamine and tyramine have differential modulatory influences on the operation of the CPGs for walking and flight in locusts.

Octopamine was found to increase the probability that the natural releasing stimulus (wind) induced a flight motor response in deafferented locust preparations. An analogous effect has been reported previously for moths [57], [58], locusts [5]; [47], [48], cockroaches [59] and fruit flies [54]. While the concentration of octopamine required to initiate flight was extraordinarily high (100 mM see also [20]), promotion of wind-induced flight by octopamine was achieved with a comparatively low, more physiological concentration (5 mM). Due to the effectiveness of the insect blood brain barrier [60] the effective dosage reaching the neuropil will be far less. Although it is to be expected that exogenously applied tyramine can bind octopamine receptors at high concentrations (cf. [61]), we propose that octopamine selectively promotes the production of flight, since its effect was not mimicked by the closely related amine tyramine, for which dedicated receptors are known [62]. Octopamine could exert its effect at multiple sites. It is generally reputed to strengthen specific synaptic connections [63], increases neuronal responsiveness in sensory pathways and evoke plateau potentials in flight interneurons (review: [64]).

Contrasting the original claim of Sombati and Hoyle [18], octopamine is unlikely to be involved in recruiting walking activity. Although octopamine application led to activation of leg motor units, these mostly fired in phase with the ongoing flight rhythm, and a walking rhythm was rarely observed, even at the highest concentrations tested (100 mM). Coupling of leg motor units to the flight motor pattern is also evident for naturally initiated flight by wind, whereby coupling was tightened in the presence of octopamine and loosened by its antagonist epinastine, both at low concentrations (5 mM, respectively 1 mM). We speculate that this may be a further selective effect of octopamine, since neither tyramine, nor its antagonist yohimbine had any influence on coupling.

Tyramine, as opposed to octopamine, readily evoked fictive walking at comparatively low concentrations (10 mM), and this could occur together with fictive flight, but only at tenfold higher concentration. Notably, and again in contrast to octopamine, leg motor units were at best only weakly coupled to the flight rhythm. While tyramine, at high concentration at least, appears to have a suppressing effect on flight initiation in Drosophila [54], we found no effect of this amine or its antagonist on the effectiveness of the wind stimulus to evoke flight. Furthermore, while in moths tyramine is reported to selectively increase the number of depressor spikes per cycle and decrease the depressor phase [50], we found no such effect of tyramine or its antagonist yohimbine in locusts. Species specific differences in the action of amines on insect motor systems accordingly seem likely.

### Final Conclusions

The finding that the networks for walking and flight which occur in the same ganglia can operate at the same time even though the behaviors are mutually exclusive, suggest that the CPG kernels are largely independent, and do not inhibit each other. Nonetheless, elements of the walking motor system can be recruited by and temporally coupled to flight, whereas the CPG for walking appears not to influence flight. Activation of these two CPGs is likely to be primarily via a cholinergic mechanism, while biogenic amines can influence the probability of activation, details of pattern structure and the degree of coupling between the two circuits. Octopamine and tyramine, two closely related amines, have differential effects on walking and flight. Taken together it appears that tyramine promotes walking activity, while octopamine promotes activation of the flight circuitry, and recruitment of leg motor units in the flight motor score. This supports the notion that octopamine and tyramine act as independent signaling molecules with dedicated receptors [65], [21], [66]. The only identified candidate neurons for aminergic modulation of the walking and flight CPGs are the population of efferent dorsal unpaired median neurons in the thoracic ganglia (review: [52], [67]). Different subsets of these neurons are selectively excited during walking and flight related activity [68]-[70], and the population as a whole express both octopamine and tyramine immunoreactivity [71]. There is however immunocytochemical evidence for tyramine containing neurons which apparently do not express octopamine in the insect brain [72], [73]. The intriguing question is whether any of these cells actual modulate the operation of CPGs for insect locomotion, and if so under which conditions, if any, they release octopamine and or tyramine?
